# Development of a Material Design Space for 4D-Printed Bio-Inspired Hygroscopically Actuated Bilayer Structures with Unequal Effective Layer Widths

**DOI:** 10.3390/biomimetics6040058

**Published:** 2021-10-06

**Authors:** Friederike Krüger, Rebecca Thierer, Yasaman Tahouni, Renate Sachse, Dylan Wood, Achim Menges, Manfred Bischoff, Jürgen Rühe

**Affiliations:** 1 Laboratory for Chemistry and Physics of Interfaces, Department of Microsystems Engineering, University of Freiburg, Georges-Koehler-Allee 103, 79110 Freiburg, Germany; ruehe@imtek.uni-freiburg.de; 2 Institute for Structural Mechanics, University of Stuttgart, Pfaffenwaldring 7, 70550 Stuttgart, Germany; bischoff@ibb.uni-stuttgart.de; 3 Cluster of Excellence Integrative Computational Design and Construction for Architecture (IntCDC), University of Stuttgart, Keplerstraße 11, 70174 Stuttgart, Germany; yasaman.tahouni@icd.uni-stuttgart.de (Y.T.); dylan.wood@icd.uni-stuttgart.de (D.W.); achim.menges@icd.uni-stuttgart.de (A.M.); 4 Institute for Computational Design and Construction (ICD), University of Stuttgart, Keplerstraße 11, 70174 Stuttgart, Germany; 5 Institute for Computational Mechanics, School of Engineering and Design, Technical University of Munich, Boltzmannstraße 15, 85748 Garching b. München, Germany; renate.sachse@tum.de

**Keywords:** material development, biomimetic bilayer actuators, hygroscopic actuation, 4D-printing, mechanical modeling

## Abstract

(1) Significance of geometry for bio-inspired hygroscopically actuated bilayer structures is well studied and can be used to fine-tune curvatures in many existent material systems. We developed a material design space to find new material combinations that takes into account unequal effective widths of the layers, as commonly used in fused filament fabrication, and deflections under self-weight. (2) For this purpose, we adapted Timoshenko’s model for the curvature of bilayer strips and used an established hygromorphic 4D-printed bilayer system to validate its ability to predict curvatures in various experiments. (3) The combination of curvature evaluation with simple, linear beam deflection calculations leads to an analytical solution space to study influences of Young’s moduli, swelling strains and densities on deflection under self-weight and curvature under hygroscopic swelling. It shows that the choice of the ratio of Young’s moduli can be crucial for achieving a solution that is stable against production errors. (4) Under the assumption of linear material behavior, the presented development of a material design space allows selection or design of a suited material combination for application-specific, bio-inspired bilayer systems with unequal layer widths.

## 1. Introduction

Hygroscopically actuated movements can be observed in various plant structures. Their function reaches from protection of pollen [1] to active seed dispersal and seed burial [2,3,4] to seed protection [5,6]. Often, these movements are caused by functional bilayer mechanisms made from the combination of a restrictive passive layer and a responsive active layer, where cellulose fibers control the direction of expansion as a response to tissue hydration [5,7,8]. These swelling effects occur without the need for metabolic energy consumption and can therefore also be observed in dead tissues [6]. These attributes have raised the interest of similarly functioning biomimetic bilayer systems in application fields such as sensor technology [9], medicine [10], and architecture [11,12,13]. Deforming bilayer structures have been built from a variety of materials such as hydrogel [14,15], wood veneer [11,12], or fiber composites [16,17]. 

More recently, 4D-printing has been used to develop structures with programmable hygroscopic movement. Using the fused filament fabrication (FFF) method, bending motion analogous to that of the biological role models can be achieved by an anisotropic arrangement of swelling fibers of a commercial wood-filled filament [18,19,20]. 

For most of these material systems, the influence of basic geometric changes such as layer thickness are well understood, enabling computational reproduction of observed curvatures based on Timoshenko’s model for bimetal bending, adapted for hygromorphic materials [12,16,17,21,22,23]. This knowledge allows for designing new demonstrators with planned and timed curving behavior [18]. However, if active and passive layer effective widths differ, e.g., due to variable spacing between adjacent printing paths or the width of the 3D-printed strands, Timoshenko’s formula has to be modified. Furthermore, to our knowledge, the influence of material parameters on curvature has not yet been analyzed systematically. Such information enables a more application-oriented material selection or development. Fields in which bilayer systems have to be designed for more than the resulting curvature would especially benefit, e.g., as in architectural applications. Here, not only the curvature of a structure due to swelling or shrinking has to be considered for a successful application, but also its deflection under self-weight, absorbed water, or wind, depending on the arrangement and support conditions of the system. Easily applicable models and measurements are favorable, because complicated modeling and simulation and extensive experimental parameter measurements are time and material consuming. To focus on the application of bio-inspired bilayer structures that aim to perform repetitive hygroscopically actuated motions, the choice of an elastic material is crucial. Therefore, Timoshenko’s simple model [23] is chosen as a starting point.

In this paper, Timoshenko’s model [23] is adapted to account for unequal effective widths of the active and passive layer. The model is validated by means of an established hygromorphic, 4D-printed bilayer system, where swelling strains and Young’s moduli of the used materials are obtained by simple, single-material experiments. For validation, various experiments on bilayers are conducted and compared to the computed values. Based on the adapted model and on basic linear bending theory, the interplay of various material parameters on curvature due to changes in moisture content and on deflection due to self-weight, including absorbed water, will further be analyzed. This is then used for the development of a material design space with fixed geometric parameters and variable values for Young’s moduli, swelling strains, and self-weights.

## 2. Materials and Methods

### 2.1. Development of Mechanical Models

We used a modeling approach for predicting the curvature due to changes in moisture content of bilayer cantilevers with unequal effective layer widths that establishes an extension of Timoshenko’s model for composite beams [23]. Considering the thicknesses *t*_a_, *t*_p_ and Young’s moduli *E*_a_, *E*_p_ of the active and passive layer, respectively, and the total thickness *h = t*_a_ *+ t*_p_ of the beam, Timoshenko’s equation provides an analytical solution for the curvature radius *r* and the curvature *κ* caused by a swelling strain *ε*_swell_ in the longitudinal direction in the active layer.

**Figure 1:**
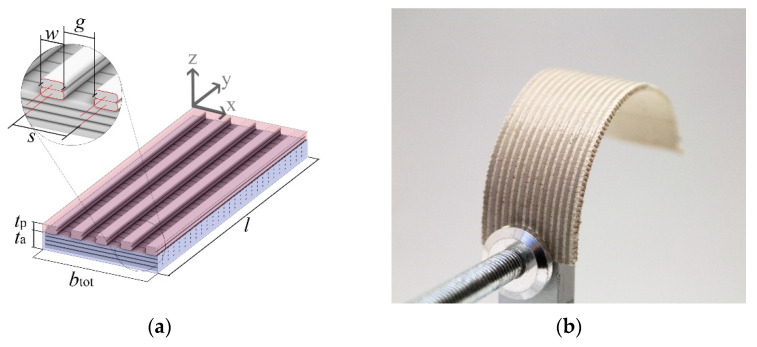
Geometry of bilayer samples. (**a**) Schematic of bilayer sample consisting of active (blue) and passive (red) layer. *l*: Length of bilayer. *t*_p_: Thickness of passive layer. *t*_a_: Thickness of active layer. *w*: Width of printed strands (extrusion width). *g*: Gap between adjacent strands. *s*: Spacing between adjacent printing paths. Effective width of active layer *b*_a_ = *b*_tot_. Effective width of passive layer was calculated as *b*_p_ = *b*_tot_ − *n*_gap_ ∙ *g*, with number of gaps *n*_gap_. Filament deposition along the *x*-axis was defined as transversal, along the *y*-axis as longitudinal. Single-material layers of the same geometry were used as samples for measurements of Young’s moduli. (**b**) Photograph of bilayer sample in dry and therefore curved state. Grey strands of passive material are visible.

To account for unequal effective widths of the active and passive layer, caused by the porous mesostructure of the 3D-printed layers, we incorporated the effective layer widths *b*_a_, *b*_p_ into the equation. The effective layer widths *b*_a_ and *b*_p_ were obtained by subtracting the width of gaps *g* between adjacent printing strands in the longitudinal direction from the total width of the respective layer and thus only considering the materialized part of the total width (Figure 1a). The analytical solution for the curvature then reads:

(1) $$\kappa =\frac{1}{r}=\frac{6\varepsilon_{\mathrm{swell}}{\left(1+m\right)}^{2}}{h\left(3{\left(1+m\right)}^{2}+\left(1+nmq\right)\left(m^{2}+\frac{1}{nmq}\right)\right)}\;,\;\mathrm{with}\;n=\frac{E_{p}}{E_{a}}\;,\;m=\frac{t_{p}}{t_{a}},\;\mathrm{and}\;q=\frac{b_{p}}{b_{a}}\;.$$

A complete derivation of the formula can be found in Appendix A. Referring to laminate theory, this model resembles the classical laminate theory (CLT), where material fibers normal to the mid-surface remain straight, normal and unstretched during deformation. Transverse shear effects are therefore excluded. The formulation can handle large rotations, but is restricted to small strains.

For the deflection of the cantilevers under self-weight, including absorbed water, Bernoulli’s geometrically linear static bending theory of beams was used. The cross-sectional porosity of the bilayers was taken into account by using effective layer widths to calculate the ideal bending stiffness of composite beams.

### 2.2. Measurements of Established System’s Parameters

#### 2.2.1. Sample Production

Laywood meta 5 filament (LayFilaments, Cologne, Germany) from two different batches was used as active layer material and combined with a generic PLA filament (SUNLU, Zhuhai, China) as passive layer material to print multi-material bilayer structures via fused filament fabrication (FFF). Both materials were printed on a dual-extruder FFF 3D-printer (FELIX TEC 4, FELIXprinters, IJsselstein, The Netherlands) equipped with two 0.5 mm brass nozzles and a heated bed. A custom toolpath design and G-code generation workflow, which was built in Rhinoceros 3D CAD environment and Grasshopper Plugin (McNeel and Associates), was used to generate digital designs and G-codes (Voxel2GCode) for 3D-printing [18].

In all samples, the printing parameters, including nozzle temperature, bed temperature, *E*-value (volume of extruded filament per mm), material layer height, and feed rate was kept constant (Table A1). By fixing these parameters, it was assured that the width of a single 3D-printed strand *w* was kept constant. The effective layer width *b* could then be controlled by the toolpath spacing parameter *s*, which is defined as the distance between the two adjacent printing toolpaths (Figure 1a) and controls the width of the gap *g* between two adjacent strands. In all active layers, 3D-printed strands were oriented transversally and in passive layers longitudinally. In all samples, active layers were printed with a spacing of 0.5 mm, resulting in a solid layer. Passive layers were printed with a spacing of 1.5 mm (except those of experiment C). Samples with different thicknesses in the active (experiment A) and passive (experiment B) layer were manufactured by varying the number of printed material layers while keeping a constant material layer height of 0.2 mm. In experiment A the number of active material layers was chosen as 2, 4, 6, 7 and 8. In experiment B the number of passive material layers was chosen as 1, 2, 4, 6 and 8. To analyze samples with different effective passive layer width (experiment C), spacing between passive layer strands was chosen as 0.5 mm, 0.6 mm, 1.0 mm, 1.5 mm and 3 mm. For each experiment, seven samples were printed and tested.

Following the manufacturer’s instructions, samples with Laywood meta 5 were rinsed for five days after printing and dried at 30% humidity for another two days to reach their responsive state. After this treatment, a repeatable response to water submerging and drying was assumed, and all samples were successively investigated in wet, humid, and dry state. For that, samples were placed in a climate chamber (KBF 115, Fa. BINDER GmbH, Tuttlingen, Germany) at 23 °C in water submersion for wet, in 80% ambient humidity for humid and in 30% ambient humidity for dry state over two days to reach equilibrium.

#### 2.2.2. Measurements of Young’s Modulus and Swelling Strains

According to Equation (1), Young’s moduli of active and passive layers *E*_a_ and *E*_p_ and the swelling strain *ε*_swell_ have to be determined for the curved humid and dry states. Additionally, for the deflection due to self-weight, Young’s moduli in wet state are required.

Young’s moduli were determined from deflections of eight monolayer samples for Laywood meta 5, tested repeatedly in all three states, and eight monolayer samples of PLA for each state. Laywood meta 5 samples were 75 mm × 20 mm × 0.8 mm with transverse filament deposition and PLA samples were 75 mm × 20 mm × 0.2 mm with longitudinal filament deposition, identical to the printing direction in the bilayer structures (Figure 1a). Length, width, thickness and weight of these samples were measured in all states to calculate the swelling strain in all directions and self-weight in all states, using a digital caliper with an accuracy of 0.02 mm and a scale with an accuracy of 0.001 mg.

#### 2.2.3. Bilayer Deflection and Curvature Measurements

**Figure 2:**
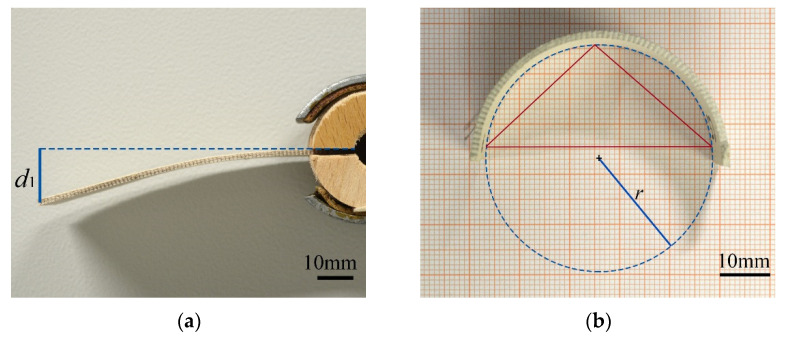
Evaluation of deflection and curvature. (**a**) Deflected sample in wet state. Measurement of deflection as the vertical line between sample base and tip; (**b**) curved bilayer sample in dry state. Evaluation of curvature by selecting three points on the sample, getting the defined triangle and calculating the radius of its circumcircle.

Deflection under self-weight and curvature upon humidity change were measured on 3D-printed bilayer samples (Figure 2). Deflection under self-weight was measured in wet state. Samples were clamped at their base for 3 mm and images were taken perpendicular to their edge (Figure 2a). Images were then analyzed with ImageJ [24,25] and deflection was measured as the vertical distance between the clamped surface and the tip of the beam. To compensate for initially slightly curved sample geometries, deflections were measured with passive layers facing upward as well as downward and their mean values were taken.

Curvatures were measured in humid and in dry state. Samples were positioned on a flat surface, standing parallel to their strong axis to avoid additional bending due to self-weight (Figure 2b). Images were taken from above and analyzed with a script in ImageJ, calculating the radius *r* of the circumference of a circle defined by three points, selected on the sample edge [24,25,26].

After measuring deflection and curvature, all bilayer samples were again submerged in water, their layers deconstructed and geometry changes of resulting monolayers were again measured in wet, humid, and dry state.

## 3. Results

### 3.1. Material Parameters of the Established Bilayer System

Values for Young’s moduli are very different between Laywood and PLA with the ratio of Young’s moduli *n* in the range of 25 and 60 for dry and humid state, respectively (Table 1). For Laywood, an approximately 10-fold increase from wet to dry state can be observed, while the Young’s moduli for PLA show a small increase from wet to dry state.

**Figure 3:**
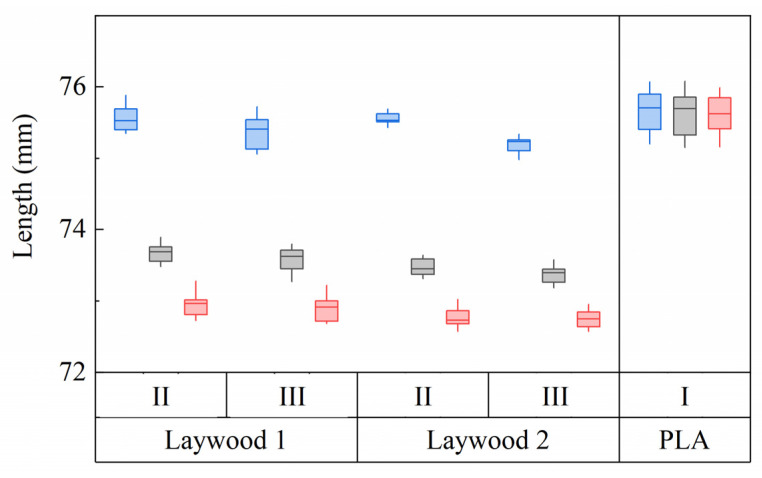
Length development of repeatedly tested samples of Laywood meta 5 and PLA. Blue: wet state. Gray: humid state (80% RH). Red: dry state (30% RH). Roman numerals refer to rinsing cycles. Laywood 1 and 2 refer to different batches of material. No differences can be seen between the second and third rinsing cycles in Laywood. Only Laywood samples show a major length change between humidity levels. *N* = 8.

Generally, no major differences between lengths in the second and third rinsing cycle could be found in monolayer samples of both Laywood batches (Figure 3). An exception was the length of wet samples of batch 2, where median lengths of 75.5 mm in the second and 75.2 mm in the third rinsing cycle were observed. As the following bilayer measurements were taken in the second rinsing cycle, these values were taken for further calculations. Length changes for all samples were calculated, defining the wet state as 100%, leading to a shrinkage of *ε*_swell_ = 2.6% from wet to humid and 3.5% from wet to dry state for batch 1 (2.8% and 3.7% for batch 2, respectively). For PLA no shrinkage could be observed (Figure 3).

### 3.2. Comparison of Measured Bilayer Deflections and Curvatures with Computed Values

Deflections under self-weight were calculated in wet state, including the absorbed water, according to Bernoulli’s geometrically linear static bending theory of beams. All curvatures due to hygroscopic swelling were modeled for the transition from wet to humid and wet to dry state, using the modification of Timoshenko’s formulation (Equation (1)).

**Figure 4:**
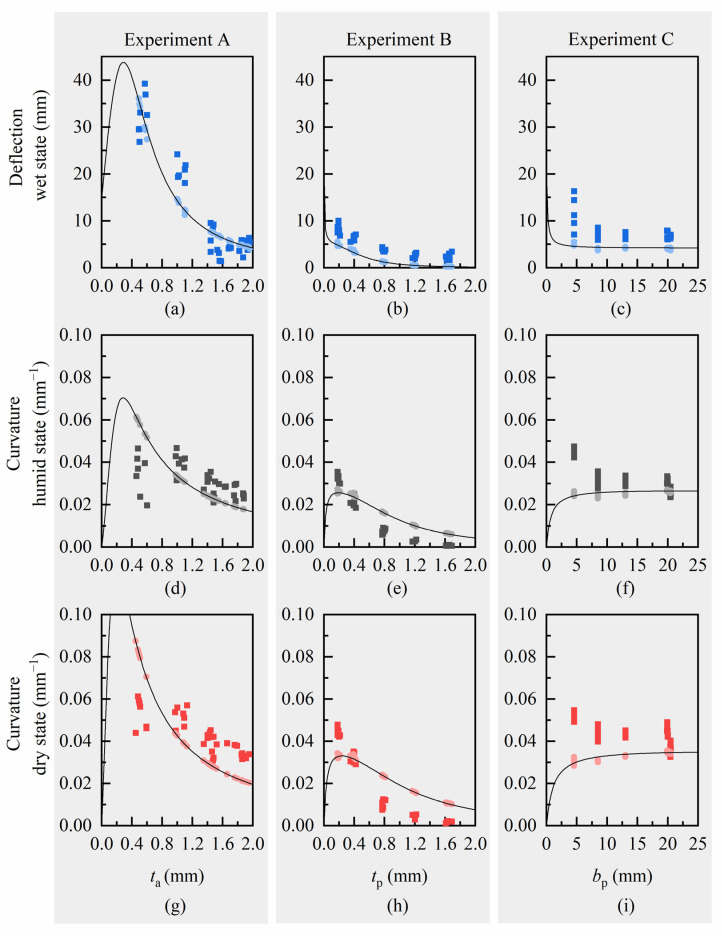
Measured and computed values for deflection and curvature of Laywood/PLA bilayers. (**a**–**c**) Blue: Deflection in wet state; (**d**–**f**) Grey: Curvature values in humid state; (**g**–**i**) Red: Curvature values in dry state. Dark squares: measured values. Light circles: computed values using the analytical models and geometric input values as measured for every individual sample. Lines represent the solution of the analytical models for a hypothetical bilayer cantilever with median values of measured geometries. All computed deflections are in good accordance with the measured values. For increasing thicknesses of active and passive layers, the computed curvatures show similar trends to the measured values with slight differences in absolute numbers. For increasing passive layer effective width, the computed and measured curvatures show opposing trends.

Deflections and curvatures were calculated for every tested sample of experiments A (varying active layer thickness), B (varying passive layer thickness) and C (varying passive layer effective width), using individual geometry input data from respective measurements in wet, humid, or dry state. For the active material, we used median Young’s moduli and swelling strains of Laywood batch 1 for experiment A and median values of Laywood batch 2 for experiments B and C. For the passive material of all samples, we used median Young’s moduli of PLA, respectively. Calculated and measured deflections and curvatures for every sample are compared and analytical curves for a hypothetical, median-based cantilever are used to broaden the range of the respective free variable (Figure 4). This analytical solution for the deflections and curvatures is given for a hypothetical cantilever, which is defined by median values of the measured geometric and material parameters (Table A2 and Table A3). For all computed values, there is no major difference between using sample-wise measured geometric input values or using the geometry of the hypothetical, median-based cantilevers. The computed deflections are all in good accordance with the measured values (Figure 4a–c). Although the absolute values differ slightly, the trends of a decreasing curvature for increasing layer thicknesses are clearly visible for both measured and calculated values in experiments A and B (Figure 4d,e,g,h). In experiment A, the measured values show a high variation, especially for thin active layers (Figure 4a,d,g). For experiment C, an opposing trend is predicted by the curvature calculation than can be seen in the measured curvature values (Figure 4f,i).

## 4. Discussion

### 4.1. Evaluation of Computed Deflections and Curvatures

As can be seen in Figure 4, the overall trends for most of the computed deflections and curvatures match the experimental data. However, for experiment C with modification of the effective width of the passive layer *b*_p_ the analytical model predicts an opposite trend for the curvature. Additionally, all values show slight variations between measured and calculated results. To examine the potential of the modified model in Equation (1) to predict the experimentally measured curvature, we fit the swelling strain *ε*_swell_ and the ratio of Young’s moduli *n* to the measured curvature values of the experiments by least squares approximation. Thus, we were able to separate effects from strongly scattering material parameters from our validation of the structural model. Here, we used again the hypothetical, median-based cantilever geometries together with the medians of measured curvatures (Table 2).

**Figure 5:**
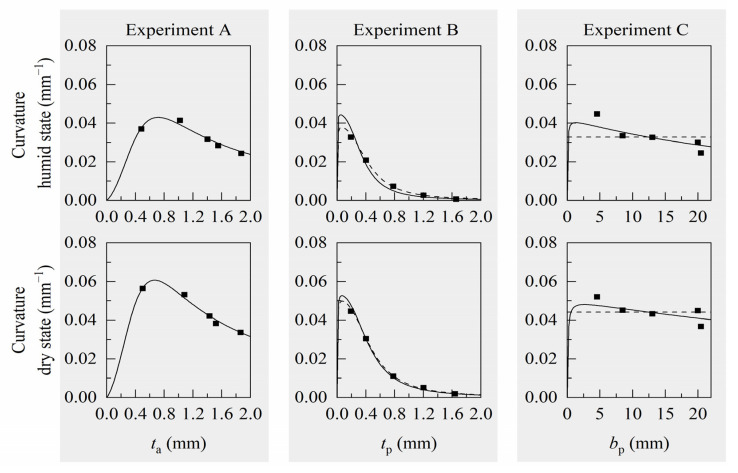
Analytical curves with optimized ratios of Young’s moduli and swelling strains. Squares: Median values from experiments. Solid line: Using optimized parameters for the modified model. Dashed line: Using optimized parameters for Timoshenko’s original model. For Experiment A with different active layer thickness *t*_a_, lines from optimizations are congruent. Both models are able to depict the measured trends of experiments A and B, while only the modified model is able to reproduce a decreasing trend of curvature for increasing passive layer effective width *b*_p_ in experiment C.

As the samples of experiment A were produced from a different batch of Laywood material than in experiments B and C, we performed separate optimizations (Table 2). A fit of parameters for Timoshenko’s original model, which assumes the equality of layer widths for the curvature calculation, was done for comparison (Table 2). With these resulting material parameters, both models are able to depict the measurements of experiments A and B, while only the modified version can reproduce the measured decreasing trend of curvature in experiment C with increasing passive layer effective width *b*_p_ (Figure 5).

The resulting elongations *ε*_swell_ from the optimization approach (Table 2) are slightly larger than those measured in single-material experiments. These differences can be explained by inaccuracies of length measurements in wet state. The extremely soft material might have been compressed during the manual measurements, resulting in an underestimation of wet sample length. The resulting ratios of Young’s moduli *n* from the optimization approach differ strongly from those measured in all single-material tests, where we found ratios of about *n* = 40. This could be due to a violation of the assumption of linear material behavior, which is a major element of Timoshenko’s model [23]. Tensile tests showed the end of the elastic range at approximately 2% for Laywood in humid and in dry state (Supplementary Materials File S1). Additionally, in single-material deflection tests of humid and dry state, a maximum elastic strain of only approximately 0.1% could be calculated (Supplementary Materials File S2), while for curved bilayers a maximum elastic strain of about 5% resulted from calculations using the modified model, which means an exceedance of the elastic limit (Supplementary Materials Files S3 and S4).

**Figure 6:**
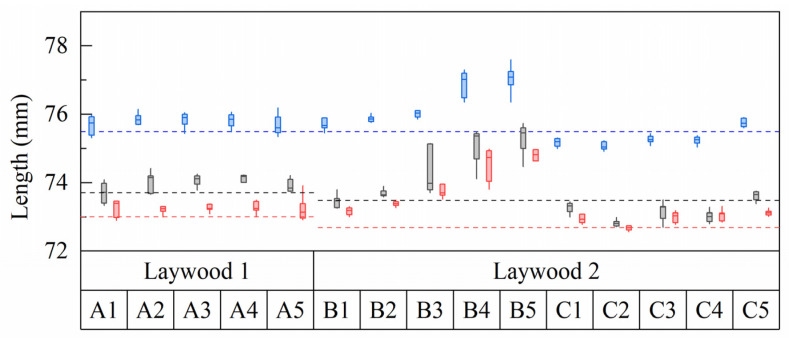
Length development of Laywood meta 5 monolayers from deconstructed bilayers. Blue: wet state; black: humid state; red: dry state; A1 to A5: increasing active layer thickness; B1 to B5: increasing passive layer thickness; C1 to C5: decreasing passive layer width. Laywood 1 and 2 refer to different batches of used Laywood meta 5 filament. Dashed lines mark the median length of pure Laywood meta 5 monolayers. With increasing passive layer thickness, a distinct increase in active layer length is visible. *N* = 7.

Assuming non-linear material behavior and therefore redefining *n* as the ratio of stiffnesses between passive and active material, changes between this ratio from *n* = 40 at elastic strains of 0.1% to *n* = 600 at strains of 5% could occur, if the materials showed softening effects for high strains. A strong hint towards plastic deformations in the active layer can be found when reviewing the length development of deconstructed bilayers: If deformations would only occur in the elastic range, lengths of deconstructed Laywood monolayers should be the same as in single layers of Laywood. This expected behavior can be seen for deconstructed Laywood layers from experiments A and C (Figure 6). However, in experiment B a strong increase in length could be observed, suggesting that plastic deformations occur when the passive layer is relatively thick and the bilayers experience rather a low curvature but high elastic strains in the active layer (Supplementary Materials File S4). Such plastic deformations should be avoided, e.g., by choosing a different material for the active layer, if the bilayer structure is intended to perform repetitive hygroscopically actuated motions.

Although the observed experimental values could not be reproduced computationally, because the potentially non-linear material behavior is not accounted for in the model, we could show that by extending the original model of Timoshenko by the effect of individual effective layer widths, a tool for the curvature prediction of bilayer cantilevers with unequal effective widths, e.g., due to 4D-printing, is provided. We therefore use the modified Timoshenko model in the following to build the material design space.

### 4.2. Development of a Design Space

To allow suitable material selection, we consider the relation between curvatures of bilayers due to humidity changes and their deflections under self-weight. Large curvatures in dry state are accompanied by large deflections in wet state. Combinations of thin active and passive layers lead to an increasing curvature, but also result in very large deflections (Figure 4a,g). An increase in passive layer effective width decreases deflection, but inhibits bending as well (Figure 4b,h). Consequently, if only geometric parameters of a bilayer system can be changed, a trade-off between large curvature and limited deflection has to be made with respect to the desired use of the bilayer structures. A good starting point for this trade-off can be found by using the analytical formulas for curvature (Equation (1)) and deflection to define a design space. To do so, all geometric parameters have to be set.

**Figure 7:**
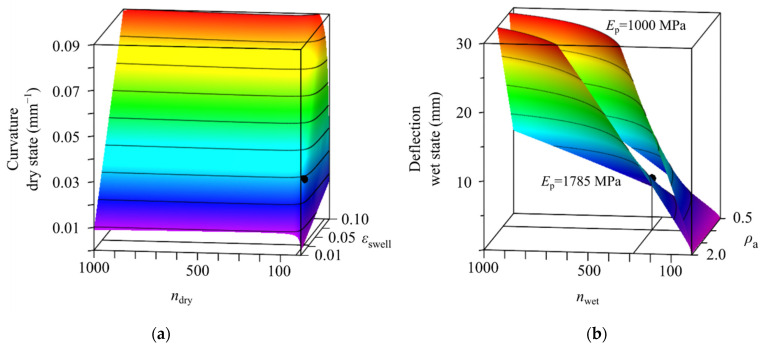
Exemplary design space for the choice of parameters of an active material for a given geometry and different assumptions on the passive material. (**a**) Solution surface of curvature for various swelling strains *ε*_swell_ and ratios of Young’s moduli *n*. Colors indicate magnitude of curvature. (**b**) Solution surfaces of deflection for various densities *ρ*_a_ and ratios of Young’s moduli *n*. The lower surface corresponds to an absolute value of the passive layer’s Young’s modulus *E*_p_ = 1785 MPa, the upper surface corresponds to *E*_p_ = 1000 MPa. For both surfaces the density of the passive material is chosen as *ρ*_p_ = 1.43 mg/mm^3^. Colors indicate magnitude of deflection. Black dots mark the solutions for the given Laywood/PLA bilayers of samples from experiment A with *t*_a_ = 1.43 mm in dry and *t*_a_ = 1.45 mm in wet state.

For an exemplary design space, we set the geometric parameters as measured for samples of experiment A in dry and in wet state with a median active layer thickness of *t*_a_ = 1.43 mm in dry and *t*_a_ = 1.45 mm in wet state (Figure 7). The density of the passive material is chosen as measured with *ρ*_p_ = 1.43 mg/mm^3^ and the passive layer’s Young’s moduli *E*_p_ = 1785 MPa (lower surface in Figure 7b) and *E*_p_ = 1000 MPa (upper surface in Figure 7b) are considered.

The solution surface for the curvature space shows a distinct kink at around *n*_dry_ = 30. Below this ratio, small changes of *n*_dry_ lead to drastic changes in the resulting curvature. The swelling strain, however, has only a linear influence on the curvature. When analyzing deflections, the active layer’s density *ρ*_a_ and *n*_wet_ have an almost linear influence on the deflection. It can be lowered by decreasing either *ρ*_a_ or *n*_wet_. Another option would be the simultaneous increase of *E*_p_ and *E*_a_.

### 4.3. Usage and Limitations of Material Design Space for Material Selection or Development

After formulating the analytical solutions for the individual material design space, one target value each for curvature and deflection can be chosen. We recommend using the target curvature to choose values for *n*_dry_ and swelling strains. Here, attention has to be drawn to existing kinks in the solution surfaces, as can be seen in the exemplary solution in Figure 7a. The values should not be chosen too close-by to achieve a stable prediction, if the material parameters used later for the actual bilayer system show typical scattering due to production errors. If commercially available or already developed materials are used, the corresponding value *n*_wet_, which will differ from *n*_dry_, if material’s stiffnesses are moisture dependent, can be calculated. If not, we recommend an educated guess to determine the range of *n*_wet_. With the set value of *n*_wet_, absolute values for *E*_a_ and *E*_p_ can be chosen from the analytical solution for deflections, leading to a resulting density of the active material. With these values at hand, it is possible to reach the target values for curvature and deflection. Additionally, further fine-tuning or variation of curvature and deflection can be realized by reiterating on the geometric parameters, such as layer thicknesses and spacing. This can also be useful to program the timing of the curvature process [18], which is so far not included in the presented mechanical model and material design space.

After this step, additional considerations are necessary. First, the justification of all important assumptions has to be checked. As could be seen, non-linear material behavior at the maximum strain resulting from curvature or deflection, can lead to differences between measured and calculated values. A more accurate modeling approach including FEM simulations would be needed (Appendix D). However, such an approach lacks an analytically closed solution and cannot be easily used to build a material design space as proposed. Additionally, if the chosen material has a high density or low stiffness in the curved state, additional deflections have to be considered in a combined load case of self-weight and hygroscopic swelling.

Other limitations can be system inherent, e.g., minimum material layer heights or the stepwise material layer height increase resulting from 3D-printing. Another effect is delamination events, which are pronounced in the presented system for passive layer thicknesses beyond 1.2 mm and passive layer widths below 4.5 mm.

Possible other limitations and the applicability of the proposed material design space will have to be investigated in future work. Another field of further research could include the timing of the deformation process into the mechanical model.

In conclusion, the curvature of the mesoporous printed bilayers can be predicted by a slightly modified Timoshenko model, which uses the effective widths of the active and passive layers. A proper choice of a suitable material combination for the generation of actuatable bilayer structures can be accelerated by developing a specific design space that is based on these simple analytical formulas. Especially the influence of small differences in the material parameters on the resulting geometry can be identified and taken into account when developing new materials.

## Figures and Tables

**Table 1:** Young’s moduli in all states. Measurements are given as median (IQR). Values were recalculated from deflections under self-weight. *N* = 8.

	Young’s Modulus in MPa		
	Wet	Humid	Dry
Laywood, batch 1	6.3 (0.8)	37.6 (11.4)	99.0 (12.8)
Laywood, batch 2	9.7 (0.5)	61.9 (10.5)	110 (36.9)
PLA	1785 (558)	2189 (303)	2467 (569)

**Table 2:** Results for least squares fit of material parameters *n* and ***ε*_swell_** to measured curvatures in humid and in dry state for original and modified Timoshenko model.

		Modified Model (Equation (1))		Original Model (Timoshenko)	
Experiment		*n*	*ε*_swell_	*n*	*ε*_swell_
Humid state	A	691.2	0.034	286.0	0.034
	B,C	610.4	0.043	149.2	0.037
Dry state	A	561.9	0.045	233.9	0.045
	B,C	299.7	0.051	111.6	0.048

**Figure A1:**
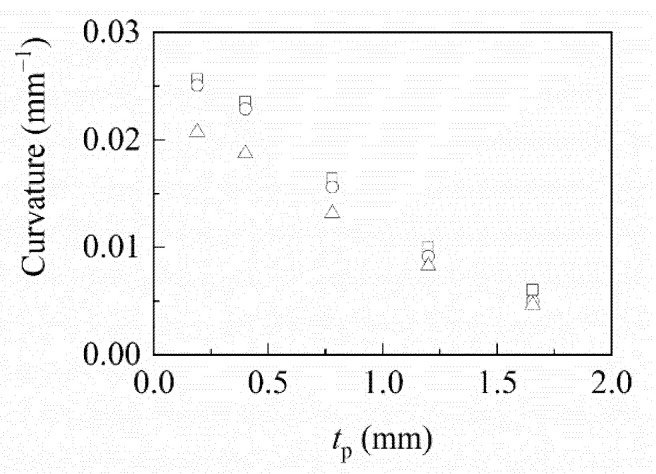
Computed curvatures for Laywood/PLA bilayers of experiment B in humid state using different modeling approaches. Open circles: Modeling and simulation based on layer-wise theory using ANSYS assuming linear elastic material. Open triangles: Modeling and simulation based on layer-wise theory using ANSYS assuming hyperelastic material. Open squares: Modeling based on classical laminate theory using the modification of Timoshenko’s model in Equation (1). For highly curved samples with compressive strains, the more realistic hyperelastic model shows an increasing difference.

## Data Availability

The data presented in this study are available in Supplementary Materials.

## Supplementary Materials

The following are available online at https://www.mdpi.com/article/10.3390/biomimetics6040058/s1, File S1: Tensile tests; File S2: Values Laywood monolayers; File S3: Bilayers strain distribution maximum; File S4: Bilayers strain distribution thick bilayers.

## Appendix A

Following Timoshenko’s analysis of bi-metal thermostats [23], we consider a bilayer strip made of one active layer that can expand due to hygroscopic swelling in the longitudinal direction and one passive layer, which will not expand due to hygroscopic changes. If the bilayer experiences changing humidity conditions and the layers are ideally bonded interlaminarly, the passive layer prevents the strip from pure expansion, leading to bending of the strip. Assuming constant swelling and constant geometric parameters along the strip, the resulting curvature *κ* will be constant. Therefore, only an infinitesimal cross-sectional element is considered. Its geometric parameters are the thicknesses and widths of the active layer (*t*_a_, *b*_a_) and passive layer (*t*_p_, *b*_p_) and the total thickness of the strip *h = t*_a_ *+ t*_p_. The material parameters are the Young’s moduli of both layers (*E*_a_, *E*_p_) and the longitudinal swelling strain of the active layer *ε*_swell_.

In case of a positive swelling elongation *ε*_swell_ in the active layer, prevention of the passive layer from pure expansion leads to an axial compressive force *P*_a_ in the active layer. As we assume no additional external force loads, static equilibrium states a tensile force *P*_p_ in the passive layer, equal in amount: $P_{a}=P_{p}=P$.

The resulting sum of the bending moments in both layers are in equilibrium with the axial forces:

(A1) $$M_{a}+M_{p}=\frac{Ph}{2}\;.$$

Assuming linear elastic material, the bending moments are related to the curvature via the bending stiffnesses:

(A2) $$M_{a}=E_{a}I_{a}\;\kappa ,\;M_{p}=E_{p}I_{p}\;\kappa \;,$$

with the respective second moment of area $I=\frac{bt^{3}}{12}$.

The assumption that fibers normal to the mid-surface, i.e., in the thickness direction, remain straight, normal and unstretched during deformation (Bernoulli’s beam theory), leads to a kinematic conclusion, where the total strains at the interface of both layers must be equal:

$$\varepsilon_{a,\mathrm{tot}}=\varepsilon_{p,\mathrm{tot}}\;.$$

For small strains, the total strains of the active layer are additively decomposed into an elastic and a hygroscopic swelling part. The passive layer only experiences elastic strains:

$$\varepsilon_{a,\mathrm{elast}}+\varepsilon_{\mathrm{swell}}=\varepsilon_{p,\mathrm{elast}}\;.$$

The elastic strains can be further split into a membrane and a bending part:

(A3) $$\frac{P_{a}}{E_{a}b_{a}t_{a}}+\frac{\kappa \;t_{a}}{2}+\varepsilon_{\mathrm{swell}}=-\frac{P_{p}}{E_{p}b_{p}t_{p}}-\frac{\kappa \;t_{p}}{2}\;.$$

Introducing the ratios $n=\frac{E_{p}}{E_{a}},\;m=\frac{t_{p}}{t_{a}},\;q=\frac{b_{p}}{b_{a}}$ and combining Equations (A1), (A2) and (A3) leads to Equation (1), i.e., the solution for curvature, valid for large rotations and small strains:

$$\kappa =\frac{1}{r}=\frac{6\varepsilon_{\mathrm{swell}}{\left(1+m\right)}^{2}}{h\left(3{\left(1+m\right)}^{2}+\left(1+nmq\right)\left(m^{2}+\frac{1}{nmq}\right)\right)}\;.$$

## Appendix B

**Table A1:** Fixed printing parameters for all samples.

	Active Layer Laywood Meta 5	Passive Layer PLA
Layer height	0.2 mm	0.2 mm
E-value	0.040	0.033
Nozzle temperature	210 °C	210 °C
Bed temperature	55 °C	55 °C
Printing speed (feed rate)	1200 mm/min	1200 mm/min

## Appendix C

Values of hypothetical, median-based cantilevers in wet (Table A2), humid, and dry state (Table A3).

**Table A2:** Hypothetical, median-based cantilever geometry and material input data for deflection calculations of bilayer experiments A, B and C in wet state.

Experiment		*t*_a_ mm	*t*_p_ mm	*b*_a_ mm	*b*_p_ mm	*L* mm	*ρ*_a_ mg/mm^3^	*ρ*_p_ mg/mm^3^	*E*_a_ MPa	*E*_p_ MPa
**wet state**	A	free	0.15	20.61	8.45	72.94	1.03	1.43	6.3	1785.4
	B	1.45	free	20.69	9.10	73.11	1.02	1.03	9.7	1785.4
	C	1.46	0.22	20.69	free	72.64	1.02	0.99	9.7	1785.4

**Table A3:** Hypothetical, median-based cantilever geometry and material input data for curvature calculations of bilayer experiments A, B and C, in humid and in dry state.

Experiment		*t*_a_ in mm	*t*_p_ in mm	*b*_a_ in mm	*b*_p_ in mm	*E*_a_ in MPa	*E*_p_ in MPa	*ε*_swell_
**humid state**	A	free	0.15	20.42	8.45	37.6	2188.7	0.026
	B	1.38	free	20.43	9.10	61.9	2188.7	0.028
	C	1.37	0.22	20.48	free	61.9	2188.7	0.028
**dry state**	A	free	0.15	20.30	8.45	99.0	2467.9	0.035
	B	1.36	free	20.36	9.10	110.4	2467.9	0.037
	C	1.37	0.22	20.37	free	110.4	2467.9	0.037

## Appendix D

Another modeling approach refers to a layer-wise theory (LWT) in finite strains. In contrast to CLT, displacements over the thickness are considered piecewise linear only within the various layers, i.e., the total displacement over the thickness can be C^0^-continuous. Additionally, transverse shear effects are considered layer-wise. We performed simulations using the commercial finite element software ANSYS (Release 20 R1, ANSYS Inc. Canonsburg, PA, USA). We modelled the bilayer cantilever beams using two stacked layers of eight-node structural solid shell finite elements (SOLSH190) that are capable of handling large rotations and large strains and we used geometrically non-linear static analysis with a load controlled path following algorithm. Convergence studies showed that a mesh of 100 elements in the longitudinal direction and 30 elements in the direction of the cantilever width are needed per layer. To account for the porous mesostructure of the 3D-printed layers, Young’s modulus of the passive layer was scaled, using the ratio of effective layer widths. The cantilever beam was clamped at one end and we applied a temperature load case, while defining the thermal expansion coefficient of the active layer in the longitudinal direction as the swelling strain *ε*_swell_. For post-processing, we defined three nodes in the longitudinal direction, at both ends and in the middle of the cantilever, all located on the mid-surface. From the displacements of these nodes, we calculated the curvature radius and the curvature of the resulting arc. For the material, we choose a linear elastic definition on the one hand and a hyperelastic Neo-Hookean material law on the other hand, using the measured Young’s moduli and a Poisson’s ratio of *ν* = 0.0.

The results for this modeling approach are exemplarily shown in Figure A1 for the bending of the median-based geometries of cantilevers from experiment B from wet to humid state (Table A3). Even for the relatively high thicknesses of active and passive layers (*t*_a_ = 1.38 mm), the differences in curvature results between the modified Timoshenko model, which corresponds to CLT, and the layer-wise modeling and simulation approach using a linear elastic material law are small. Consideration of a more realistic hyperelastic material law shows differing results for the highly curved samples that experience compressive elastic strains (Supplementary Materials File S3).
