# Supplementary material for: Development of a Material Design Space for 4D-Printed Bio-Inspired Hygroscopically Actuated Bilayer Structures with Unequal Effective Layer Widths
Source: Biomimetics (Basel). 2021 Oct 6;6(4):58. doi: 10.3390/biomimetics6040058 (PMC8544213; doi:10.3390/biomimetics6040058)
Supplement: Supplementary file 1 [file biomimetics-06-00058-s001.zip › Krueger_Thierer_et_al/S1_Tensile_tests.pdf]

Measurement of Young's modulus from tensile tests

1. Materials and methods

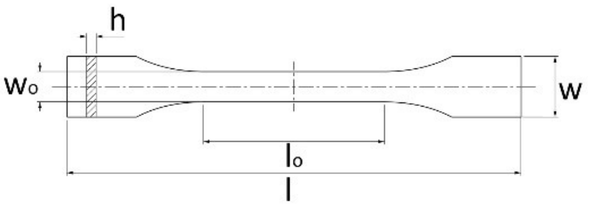

Figure 1: Sample geometry for tensile tests. Samples consisted of single material. Sample geometry corresponds to sample type 1BA of DIN EN 527with a differing thickness of 1.6 mm.

The tensile moduli of Laywood meta 5 and PLA in all three states were determined according to DIN EN 527-2 (Table 1) on eight printed tensile bars for each material and state (Figure 1) using a universal testing machine (inspect table 5, Hegewald & Peschke Mess- und Prüftechnik GmbH, Nossen, Germany). Humidity was adjusted using a temperature chamber (T56 LN2, mytron Bio- und Solartechnik GmbH, Heilbad Heiligenstadt, Germany) where water vapour or nitrogen were introduced.

Table 1: Parameters for tensile tests. Non-specified parameters were chosen in accordance with DIN EN 527. Tests were performed on a universal testing machine, using a temperature chamber to control environmental conditions.

	Laywood meta 5	PLA
**Test speed**	0.6 mm/min	0.6 mm/min
**Final Displacement**	0.87 mm (wet), 0.3 mm (dry, humid)	0.3 mm
**Load cell**	100 N (wet), 1000 N (dry, humid)	1000 N
**Printing direction**	transversal	longitudinal

2. Results

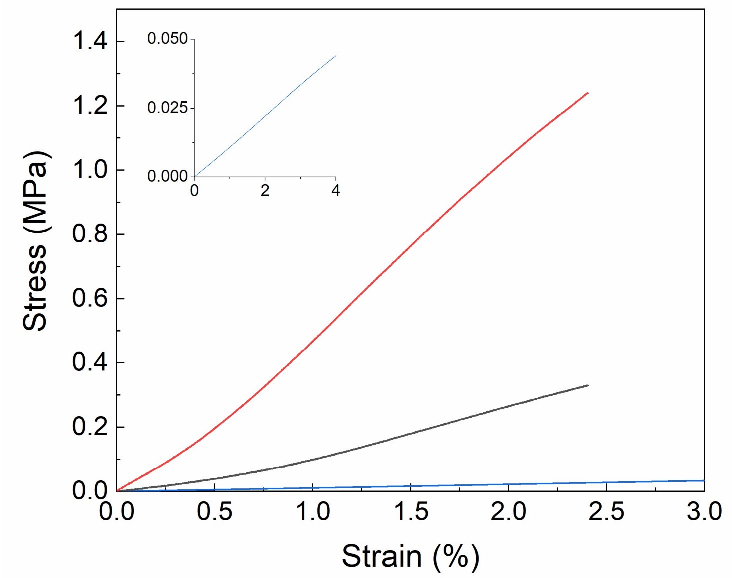

Figure 2: Exemplary curves from tensile tests for Laywood meta 5. Blue: Wet state. Green: Humid state. Red: Dry state. Young's modulus decreases with water uptake. The linear range ends at about 2% strain.

Values for Young's moduli are very different between Laywood and PLA with the ratio of Young's moduli *n* in the range between 20 and 60 for dry and humid state respectively (Table 2). For tensile moduli, an approximately 60-fold increase from wet to dry state can be seen in Laywood. The tensile moduli for PLA show a small increase from wet to dry state. Due to a more similar stress state, the flexural moduli were used for calculations of bilayer deflections and curvatures.

Table 2: Young's moduli in all states. Measurements given as median (IQR). N = 8

	Tensile modulus in MPa		
	Wet	Humid	Dry
**Filament 1**	1.2 (0.2)	17.1 (1.1)	56.8 (4.6)
**Filament 2**	0.8 (0.1)	17.9 (3.2)	63.4 (7.3)
**PLA**	1008 (19)	1078 (26)	1070 (24)
