# Supplementary material for: Development of a Material Design Space for 4D-Printed Bio-Inspired Hygroscopically Actuated Bilayer Structures with Unequal Effective Layer Widths
Source: Biomimetics (Basel). 2021 Oct 6;6(4):58. doi: 10.3390/biomimetics6040058 (PMC8544213; doi:10.3390/biomimetics6040058)
Supplement: Supplementary file 1 [file biomimetics-06-00058-s001.zip › Krueger_Thierer_et_al/S3_Bilayers_strain_distribution_maximum.pdf]

```
> restart:with(plots):
```

S. Timoshenko "Analysis of bi-metal thermostats"

Setup: bi-metal stip with thickness h, uniformly heated by DeltaT
consisting of 2 metals with coefficients of expansion alpha_T, Young's moduli E, thicknesses thk, modified by additional widths

here:
Layer 1 is passive (0 % strain), material PLA
Layer 2 is active with variable % temperature strain, material LAYWOOD meta5

EQUILIBRIUM:
of forces in layers: P_1 = P_2 (=P)
of moments in layers: P*h/2 = M_1+M_2
internal forces, no external forces

```
> P_1 := P;
  P_2 := P;
  EQU1 := M_1 + M_2 = P*h/2;
```

$$P\_1 := P$$

$$P\_2 := P$$

$$EQU1 := M\_1 + M\_2 = \frac{P\,h}{2} \quad (1)$$

MATERIAL:
M = EI * kappa = EI * 1/r

```
> MAT1 := M_1 = E_1*I_1/r;
  MAT2 := M_2 = E_2*I_2/r;
```

$$MAT1 := M\_1 = \frac{E\_1\,I\_1}{r}$$

$$MAT2 := M\_2 = \frac{E\_2\,I\_2}{r} \quad (2)$$

Combining EQU and MAT

```
> EQUMAT:=subs({MAT1,MAT2},EQU1);
```

$$EQUMAT := \frac{E\_1\,I\_1}{r} + \frac{E\_2\,I\_2}{r} = \frac{P\,h}{2} \quad (3)$$

KINEMATICS:
on the bearing surfaces the total elongation/strain of both metals must be equal
2 components of strain:
thermal strain: eps_T = alpha_T_i * Delta_T
mechanical ("elastic") strain:
due to forces: eps_F = P / EA
due to curvature: eps_kappa = kappa * thk_i / 2 = thk_i / (2r)

```
> KIN1 := alpha_T_1*Delta_T + P_1 / (E_1*thk_1*width_1) + thk_1 /
  (2*r) = alpha_T_2*Delta_T - P_2 / (E_2*thk_2*width_2) - thk_2 /
  (2*r);
```

$$KIN1 := alpha\_T\_1\ Delta\_T + \frac{P}{E\_1\ thk\_1\ width\_1} + \frac{thk\_1}{2\ r} = alpha\_T\_2\ Delta\_T - \frac{P}{E\_2\ thk\_2\ width\_2} - \frac{thk\_2}{2\ r} \tag{4}$$

Combining KIN and (EQU,MAT)

```
> P:=solve(EQUMAT,P);
  EQUMATKIN := KIN1;
```

$$P := \frac{2\ (E\_1\ I\_1 + E\_2\ I\_2)}{r\ h}$$

$$EQUMATKIN := alpha\_T\_1\ Delta\_T + \frac{2\ (E\_1\ I\_1 + E\_2\ I\_2)}{r\ h\ E\_1\ thk\_1\ width\_1} + \frac{thk\_1}{2\ r} = alpha\_T\_2\ Delta\_T - \frac{2\ (E\_1\ I\_1 + E\_2\ I\_2)}{r\ h\ E\_2\ thk\_2\ width\_2} - \frac{thk\_2}{2\ r} \tag{5}$$

Second moment of inertia

```
> I_1:=width_1*thk_1^3/12;
  I_2:=width_2*thk_2^3/12;
```

$$I\_1 := \frac{width\_1\ thk\_1^3}{12}$$

$$I\_2 := \frac{width\_2\ thk\_2^3}{12} \tag{6}$$

Resulting equation for curvature radius r

```
> r:=simplify(solve(EQUMATKIN,r));
```

$$r := \left(E\_1^2\ thk\_1^4\ width\_1^2 + 3\ thk\_1\ width\_2\ E\_1 \left(\frac{thk\_1^2}{3} + h\ thk\_1 + thk\_2 \left(h + \frac{thk\_2}{3}\right)\right) E\_2\ thk\_2\ width\_1 + E\_2^2\ thk\_2^4\ width\_2^2\right) \Big/ \left(6\ Delta\_T\ E\_1\ E\_2\ h\ thk\_1\ thk\_2\ width\_1\ width\_2\ (-alpha\_T\_1 + alpha\_T\_2)\right) \tag{7}$$

Ratio of Young's moduli n=E_1/E_2

```
> E_1:=n*E_2;
  r:=simplify(eval(r)):
```

$$E\_1 := n\ E\_2 \tag{8}$$

Ratio of thicknesses m=thk_1/thk_2 and total height h=thk_1+thk_2

```
> thk_1:=m*h/(1+m);
  thk_2:=  h/(1+m);
```

$$thk\_1 := \frac{m\ h}{1 + m}$$

$$thk\_2 := \frac{h}{1 + m} \tag{9}$$

Ratio of widths q=width_1/width_2

```
> width_1:=q*width_2;
```

$$width\_1 := q\ width\_2 \tag{10}$$

Resulting curvature:

```
> curv:=simplify(1/r);
```

$$curv := -\frac{6\ (alpha\_T\_1 - alpha\_T\_2)\ q\ m\ n\ Delta\_T\ (1+m)^2}{h\ \left(n^2\ m^4\ q^2 + 4\ m^3\ n\ q + 6\ m^2\ n\ q + 4\ m\ n\ q + 1\right)} \quad (11)$$

Timoshenko's original formula (without variable widths)

```
> curvTimo:=6*(alpha_T_2-alpha_T_1)*Delta_T*(1+m)^2/(h*(3*(1+m)^2+
  (1+m*n)*(m^2+1/(m*n))));
```

$$curvTimo := \frac{6\ (-alpha\_T\_1 + alpha\_T\_2)\ Delta\_T\ (1+m)^2}{h\ \left(3\ (1+m)^2 + (m\ n+1)\ \left(m^2 + \frac{1}{m\ n}\right)\right)} \quad (12)$$

Data Input

Bilayer experiment B1 wet-to-dry with n and alpha_T_2 from optimization

Measured values

```
> Delta_T:=1;
  alpha_T_1:=0.0;

  width_1:=9.10;
  width_2:=20.36;
  q:=width_1/width_2;

  thk_1:=0.19;
  thk_2:=1.36;
  m:=thk_1/thk_2;
  h:=thk_1+thk_2;

  curv_exp:=0.0446;

  n:=299.705696811487;
  alpha_T_2:=-0.0509093164628522;
```

$$Delta\_T := 1$$
$$alpha\_T\_1 := 0.$$
$$width\_1 := 9.10$$
$$width\_2 := 20.36$$
$$q := 0.4469548134$$
$$thk\_1 := 0.19$$
$$thk\_2 := 1.36$$
$$m := 0.1397058824$$
$$h := 1.55$$
$$curv\_exp := 0.0446$$
$$n := 299.705696811487$$
$$alpha\_T\_2 := -0.0509093164628522 \quad (13)$$

Evaluation of strains over beam thickness

Thermal strain: eps_T = alpha_T_i * Delta_T (constant over thickness)

```
> eps_Temp_1 := alpha_T_1*Delta_T;
  eps_Temp_2 := alpha_T_2*Delta_T;
```

$$eps\_Temp\_1 := 0.$$

$$eps\_Temp\_2 := -0.0509093164628522 \quad (14)$$

Mechanical/elastic strain
due to normal forces: eps_F = P / EA with A = thk_i * width (constant over thickness)
due to curvature: eps_kappa = kappa * z_i = z_i / r (linear over thickness, with coordinate z_i starting from middle of each layer downwards)

```
> eps_Mech_1F := P_1 / (E_1*thk_1*width_1);
  eps_Mech_1M_top := (-thk_1/2)/r;
  eps_Mech_1M_mid := 0;
  eps_Mech_1M_bottom := (thk_1/2)/r;
  eps_Mech_2F := - P_2 / (E_2*thk_2*width_2);
  eps_Mech_2M_top := (-thk_2/2)/r;
  eps_Mech_2M_mid := 0;
  eps_Mech_2M_bottom := (thk_2/2)/r;
```

$$eps\_Mech\_1F := -0.0006961533314$$

$$eps\_Mech\_1M\_top := 0.004558179576$$

$$eps\_Mech\_1M\_mid := 0$$

$$eps\_Mech\_1M\_bottom := -0.004558179576$$

$$eps\_Mech\_2F := 0.01302801395$$

$$eps\_Mech\_2M\_top := 0.03262696960$$

$$eps\_Mech\_2M\_mid := 0$$

$$eps\_Mech\_2M\_bottom := -0.03262696960 \quad (15)$$

Check: total strain on bearing surface (bottom passive and top active) must be equal

```
> eps_Total_1 := eps_Temp_1 + eps_Mech_1F + eps_Mech_1M_bottom;
  eps_Total_2 := eps_Temp_2 + eps_Mech_2F + eps_Mech_2M_top;
```

$$eps\_Total\_1 := -0.005254332907$$

$$eps\_Total\_2 := -0.00525433291 \quad (16)$$

Plot strains over thickness

Functions over thickness, parameter z starting from bottom upwards

```
> eps_Temp_fct:=piecewise(0<=z and z<thk_2,alpha_T_2*Delta_T,
  thk_2<=z and z<=(thk_2+thk_1),alpha_T_1*Delta_T);
  eps_Mech_F_fct:=piecewise(0<=z and z<thk_2,-P_2/(E_2*thk_2*
  width_2),thk_2<=z and z<=(thk_2+thk_1),P_1/(E_1*thk_1*width_1));
  eps_Mech_M_fct:=piecewise(0<=z and z<thk_2,-(-thk_2/2+z)/r,
  thk_2<=z and z<=(thk_2+thk_1),-(-thk_2-thk_1/2+z)/r);
```

$$eps\_Temp\_fct := \begin{cases} -0.0509093164628522 & 0 \leq z < 1.36 \\ 0. & 1.36 \leq z \leq 1.55 \end{cases}$$

$$eps\_Mech\_F\_fct := \begin{cases} 0.01302801395 & 0 \leq z < 1.36 \\ -0.0006961533314 & 1.36 \leq z \leq 1.55 \end{cases}$$

$$eps\_Mech\_M\_fct := \begin{cases} -0.03262696960 + 0.04798083764\, z & 0 \leq z < 1.36 \\ -0.06981211877 + 0.04798083764\, z & 1.36 \leq z \leq 1.55 \end{cases} \quad \textbf{(17)}$$

```
> eps_Total_fct:=eps_Temp_fct+eps_Mech_F_fct+eps_Mech_M_fct;
```

$$eps\_Total\_fct := \left(\begin{cases} -0.0509093164628522 & 0 \leq z < 1.36 \\ 0. & 1.36 \leq z \leq 1.55 \end{cases}\right) + \left(\begin{cases} 0.01302801395 & 0 \leq z < 1.36 \\ -0.0006961533314 & 1.36 \leq z \leq 1.55 \end{cases}\right) + \left(\begin{cases} -0.03262696960 + 0.04798083764\, z & 0 \leq z < 1.36 \\ -0.06981211877 + 0.04798083764\, z & 1.36 \leq z \leq 1.55 \end{cases}\right) \quad \textbf{(18)}$$

Total strains on top and bottom

```
> eps_Tot_top:=eval(eps_Total_fct,z=h);
  eps_Tot_bottom:=eval(eps_Total_fct,z=0);
```

$$eps\_Tot\_top := 0.003862026239$$

$$eps\_Tot\_bottom := -0.07050827211 \quad \textbf{(19)}$$

temperatur strain (red)

```
> plot_T:=plot([eps_Temp_fct,z,z=0..(thk_1+thk_2)],color=red,
  legend=['eps_temp']):
```

normal strain (blue)

```
> plot_F:=plot([eps_Mech_F_fct,z,z=0..(thk_1+thk_2)],color=blue,
  linestyle=dash,legend=['eps_normal']):
```

bending strain (green)

```
> plot_M:=plot([eps_Mech_M_fct,z,z=0..(thk_1+thk_2)],color=green,
  linestyle=dash,legend=['eps_bending']):
```

elastic strain=normal+bending (purple)

```
> plot_elast:=plot([eps_Mech_F_fct+eps_Mech_M_fct,z,z=0..(thk_1+
  thk_2)],color=purple,legend=['eps_elastic']):
```

total strain (black)

```
> plot_Total:=plot([eps_Total_fct,z,z=0..(thk_1+thk_2)],color=
  black,linestyle=dashdot,legend=['eps_total']):
```

display total, elastic and 3 components in one

red: temperature

blue: normal force

red: bending

purple: elastic

black: total

```
> display(plot_elast,plot_F,plot_M,plot_T,plot_Total,labels=
```

```
['strain','z']);
```

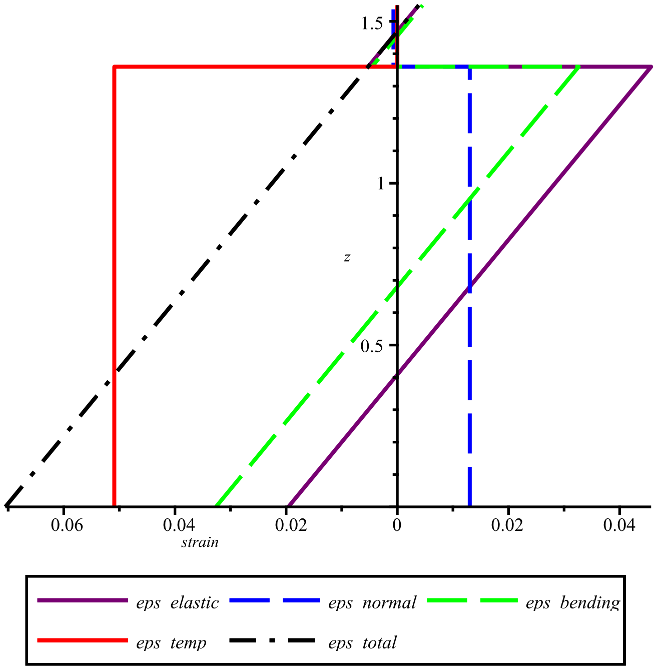

>>>Maximum elastic strain of ~ 5% at the top of the active layer

>>>Compressive strain of ~ 2% at the bottom of the active layer
